# Case report: Diverticulitis complicating a giant Meckel’s divertuculum

**DOI:** 10.1016/j.ijscr.2019.10.074

**Published:** 2019-11-01

**Authors:** Javier García-Quijada García, Carlos Bustamante Recuenco, Alberto Carabias Hernández, Ainhoa Valle Rubio

**Affiliations:** a Hospital Universitario de Getafe, Carretera Madrid-Toledo km 12.5, 28905, Madrid, Spain; b Hospital Universitario de Getafe, Madrid, 28905, Spain

**Keywords:** Diverticulum, Meckel, Giant, Abdomino-pelvic CT, Adult, Intestinal resection

## Abstract

**Introduction:**

In this paper, we discuss a unique case of diverticulitis in which the patient presented a giant Meckel’s diverticulum.

**Presentation of case:**

The patient was a 44 year old male whose medical history included a laparoscopic gastric bypass four years before the finding, and chronical high blood pressure. The patient came to the emergency department with an abdominal pain and elevated acute-phase reactants. An abdominal CT revealed a 17 cm long Meckel’s diverticulum with signs of severe inflammation. The patient was then taken to the operating room, and subjected to a 4 cm ileum resection, including the entire diverticulum, with a manual end-to-end anastomosis.

**Discussion:**

Although Meckel’s diverticulum is the most common congenital abnormality of the intestinal tract, it is unusual for it to cause symptoms in adults. However, when a patient arrives at the emergency department with a complicated Meckel’s diverticulum, an early diagnosis is essential to prevent serious complications, such as perforation of the diverticulum and subsequent peritonitis. The presence of a giant diverticulum is an extremely rare condition. There are few publications to date, but these diverticula are associated with more complications, presenting a higher risk of torsion, volvulus or intestinal obstruction.

This paper include a bibliographic review of existing studies on etiopathogenesis, and the diagnosis and treatment of complicated Meckel’s diverticulum, particularly in its giant variant.

**Conclusion:**

Although Meckel’s diverticulitis is a rare entity, it can appear as an acute abdomen. An early diagnosis and treatment to prevent subsequent complications is essential to ensure an optimal recovery.

## Highlights

• Meckel’s diverticulitis is a rare condition which may need surgical approach.
• It is uncommon that Meckel’s diverticulum causes symptomatic disease in adults.
• Although early detection is often challenging, an abdominal CT-scan may establish a certain diagnosis in giant diverticula.
• Emergency surgery is mandatory in patients with complicated Meckel’s diverticulum.
• Segmental resection is the approach of choice when a giant diverticulum has inflammatory signs.

## 1 Introduction

Meckel’s diverticulum is the most common congenital abnormality on the gastrointestinal tract, with an estimated prevalence is about 2 % [1]. It is considered a ‘true diverticulum’, as it encompasses all layers of the intestine. It is located at the antimesenteric border in the medium-distal ileum, about 100 cm above the ileocecal valve. Their size is variable, but the classical description considers diverticula bigger than 5 cm as being ‘giant diverticula’.

It is often an indolent pathology, with only 4–9 % of patients experiencing any symptoms [2,3]. Among these, children are the most prone to experiencing symptoms associated with a Meckel’s diverticulum, such as gastrointestinal bleeding, abdominal pain or intestinal obstruction. Diverticulitis resulting from a Meckel’s diverticulum is a rare condition, and it is believed that it is produced by the obstruction of the base by torsion, entheroliths or inflammatory tissue. Diverticulitis presents itself as an acute abdominal pain, and can lead to a perforation of the intestine and subsequent peritonitis.

We present the case of a 44-year-old male that was attended in an emergency department because of a complication deriving from a giant Meckel’s diverticulum. This work has been reported in line with the SCARE criteria [4].

## 2 Presentation of case

Our patient was a 44-year-old male, with a history of high blood pressure and having undergone a laparoscopic bariatric surgery 4 years before, with no complications. The patient arrived at our emergency department having experienced strong abdominal pain during a 10-day period. The pain was located in the mesogastrium, irradiating to the lumbar zone. Other symptoms described by the patient were nausea without vomiting, abdominal distension and fever of up to 38 °C. Physical examination revealed diffuse rebound tenderness with abdominal distension. An increased C-reactive protein level and leucocytosis with neutrophilia were also found in subsequent blood tests.

**Fig. 1:**
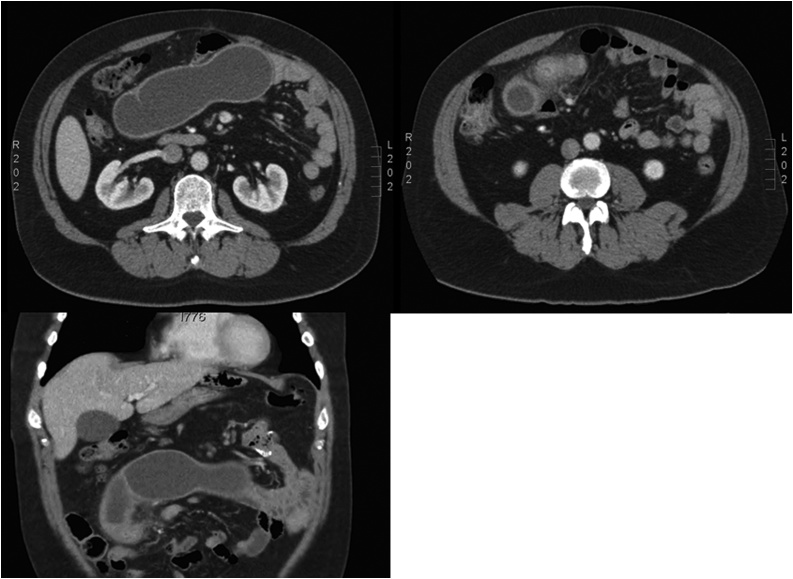
Contrast-enhanced CT-scan shows a 17 cm-long blind tubular lesion, filled with liquid contents and inflammatory changes around the base, located at proximal ileum.

A contrast-enhanced abdominal CT-scan was performed, showing findings compatible with Meckel’s diverticulitis (Fig. 1), described as a blind tubular lesion about 17 cm long, filled with liquid contents and inflammation around the base, and located in the proximal ileum.

Due to these clinical findings and the results of complementary tests, the patient was transferred to the operating room.

**Fig. 2:**
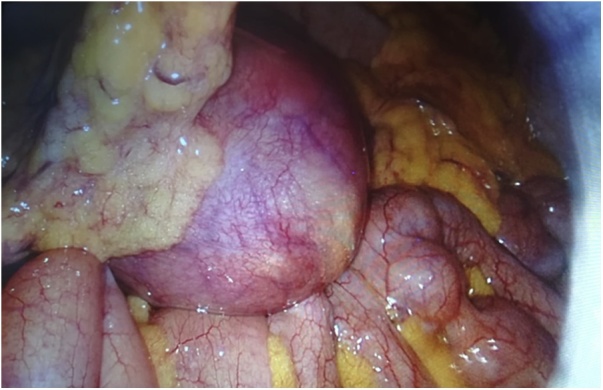
Laparoscopic approach showed an enlarged congestive saccular dilatation with severe adhesions to surrounding tissues.

A laparoscopic examination under general anesthesia revealed a large and congestive saccular dilation, adhered by inflammatory tissue to the omentum and adjacent tissues (Fig. 2).

**Fig. 3:**
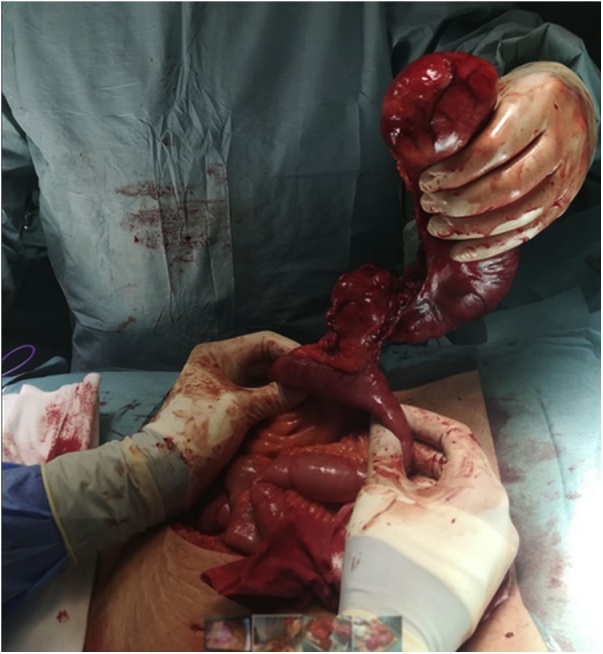
Intraoperative picture of the giant Meckel’s diverticulum.

These findings led to us converting to open surgery via midline laparotomy, finding an erythematous, hard consistency diverticulum around 18 cm long, located at the antimesenteric border of the ileum, 50 cm above the ileocecal valve (Fig. 3). The rest of the intestinal tract was normal, with no complications at the jejunojejunal and gastrojejunal anastomosis of the previous laparoscopic bypass.

A “T resection” of approximately 4 cm of ileum was performed, including the base of the diverticulum. Then, a manual end-to-end anastomosis was performed for intestinal reconstruction.

**Fig. 4:**
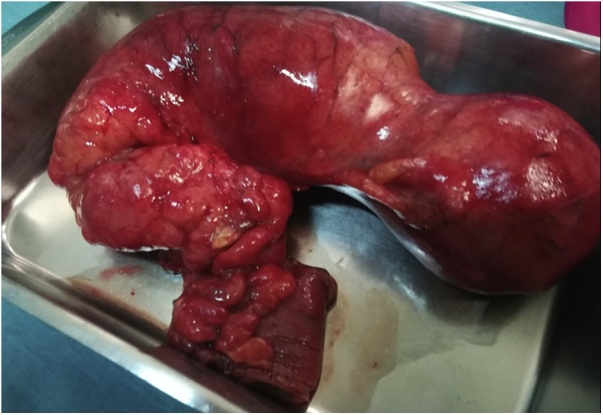
“T shape” resection specimen, including Meckel’s diverticulum and 4 cm of proximal ileum.

Anatomo-pathological analysis of the resection specimen (Fig. 4) reported a diverticulum of 15 × 6,5 × 7 cm with small intestine mucosa and acute inflammation with ulcerations, confirming the diagnosis of a giant Meckel’s diverticulum.

During the post-operatory the patient developed a paralytic ileus, managed conservatively, and a surgical site infection, managed with local wound cures. The patient was discharged in good condition after a week of hospital care.

## 3 Discussion

Although Meckel’s diverticulum is the most common congenital abnormality of the intestinal tract, it is unusual for it to cause symptoms in adults. The estimated incidence of complications associated to Meckel’s diverticulum ranges from 4 % to 9 % [3,5]. However, incidence is higher in pediatric population, with up to 60 % of cases potentially resulting in complications before reaching 10 years of age [12]. Intestinal obstruction and bleeding are the most common complications in these patients. In adults, however, intestinal obstruction and diverticulitis are more frequent than bleeding [6]. Although other complications have been described (such as umbilical alterations, parasitic diverticulum and diverticular cancer), they represent a much lower number of cases.

The presence of ectopic tissue is mainly associated with an increased risk of gastrointestinal bleeding, the presence of which is estimated to be 4.6–71 % in symptomatic patients for gastric ectopia, 0–12 % for pancreatic ectopia, and much less frequent duodenal or colic ectopia. In patients with uncomplicated diverticulum, the presence of tissue ectopia is less frequent [6].

Multiple risk factors for the appearance of complications have been described, such as male gender, age under 50 years, a diverticulum greater than 2 cm in length, or the presence of ectopic tissue. The risk of complications is estimated to be, respectively, 17 %, 25 %, 42 % and 70 % when presenting one, two, three or all four risk factors [7]. According to some authors, the presence of any of these factors would justify prophylactic resection of the diverticulum, although this opinion is still controversial, as the complication rate is rather low [3,12]. Nonetheless, all complicated diverticulum should be resected. In asymptomatic patients, treatment must be individualized, taking into account existing risk factors.

When a patient arrives at the emergency department with a complicated Meckel’s diverticulum, an early diagnosis is essential to prevent serious complications, such as perforation of the diverticulum and subsequent peritonitis. Given its low frequency and clinical variability, a successful diagnosis can be challenging.

If the patient presents a gastrointestinal bleeding, and once other causes have been excluded endoscopically, certain procedures may be useful in determining the etiology, such as an angiography [8], a double-balloon enteroscopy [9] or an endoscopic capsule [10]. The use of a Tc-99m scan has been classically described for the diagnosis of Meckel’s diverticulum, for its ability to detect gastric ectopic tissue. Although this method has an estimated sensitivity of 89.6 % and 97.1 % specificity, this test is unable to detect diverticula without tissue ectopia, which happens in a majority of cases [6].

When the patient presents symptoms more suggestive of intestinal obstruction or diverticulitis, an abdominal CT-scan can provides more information, with an accuracy of 10–50 % having been described in patients with diverticulitis or intestinal obstruction [11,14].

However, an exploratory laparoscopy, laparotomy, or endoscopic exploration is what finally confirms the diagnosis. In all patients in whom the appendix has normal characteristics during a surgical examination, the presence of a Meckel’s diverticulum that justifies the symptoms should be reviewed [12].

There is also controversy regarding which surgical technique to employ. It is accepted that a segmental “T” resection, with part of the ileum including the base of the diverticulum, ensures complete excision of the ectopic tissue and surrounding inflammation, allowing an anastomosis with healthy margins against wedge or tangential resection. Therefore, a segmental resection is recommended when palpation or abnormalities in the base are detected, or when the diverticulum has a wide neck or a diameter/length ratio less than 2, which increases the risk of incomplete excision of ectopic tissue [15].

The presence of a giant diverticulum is an extremely rare condition. There are few publications to date, but, as mentioned above, these diverticula are associated with more complications, presenting a higher risk of torsion, volvulus or intestinal obstruction [13]. In our case study, the patient also complained about severe abdominal pain, which resulted in a clear decision to perform surgical intervention. Naturally, in instances such as these, an abdominal CT-scan has a high diagnostic performance, as it allows for easy identification of any blind sac that may be present (from which data of an active infection may be inferred). It played a key role in performing an early diagnosis, reducing the risk of morbidity and mortality. Once we had a diagnosis, we performed an ileum T-resection, which, although associated with a higher risk of stenosis or wound infection [13] than simple diverticulectomy, ensures the total removal of possible ectopic mucosa that could lead to complications, and allows for a safer anastomosis on healthy tissue.

In our opinion, the laparoscopic approach offers undeniable advantages, such as a better postoperative recovery, a lower surgical wound infection rate or a lower incisional hernia rate. In our patient, the size and consistency of the diverticulum and the inflammatory adhesions preventing adequate exposure of the field made it necessary for us to convert to open surgery, which, although safer, entails a series of disadvantages associated with performing a larger incision, such as a higher risk of infection of the wound (which the patient did in fact suffer during the post-operatory period).

## 4 Conclusion

Although Meckel’s diverticulitis is a rare entity, it can appear as an acute abdomen. For this reason, this entity must be taken into account in the differential diagnosis of all patients with suspected acute abdomen, since it is a potentially serious surgical pathology with significant associated morbidity and mortality if not treated in due time. An early diagnosis and treatment to prevent the disease from progressing is essential to ensure an optimal recovery. Although Meckel’s diverticulum can be challenging to diagnose, an early performance of an abdominal CT-scan may be decisive, as was our case, allowing for the swift identification of the giant diverticulum, and permitting an early intervention to prevent the disease from progressing.

## Funding

The authors received no financial support for the research, authorship, and/or publication of this article.

## Ethical approval

Exception from ethical approval-case report only, consent from the patient provided at request.

## Consent

Written informed consent was obtained from the patient for publication of this case report and accompanying images. A copy of the written consent is available for review by the Editor-in-Chief of this journal on request.

## Author’s contribution

Mr. García-Quijada García J.: study desing. data analysis and interpretation, writing and submission of the paper.

Mr. Bustamante Recuenco C.: study desing. data analysis and interpretation, writing and submission of the paper.

Dr. Carabias Hernández A.: interpretation of data, writing the paper.

Mrs. Valle Rubio A: interpretation of data.

## Registration of research studies

NA.

## Guarantor

Carabias Hernández A.

García-Quijada García J.

Bustamante Recuenco C.

Jover Navalon JM.

## Provenance and peer review

Not commissioned, externally peer-reviewed.

## Declaration of Competing Interest

The authors declare no conflict of interest.

## References

[1] Sagar J., Kumar V., Shah D.K. (2006). Meckel’s diverticulum: a systematic review. J. R. Soc. Med..

[2] Cullen J.J., Kelly K.A., Moir C.R. (1994). Surgical management of Meckel’s diverticulum. An epidemiologic, population-based study. Ann. Surg..

[3] Soltero M.J., Bill A.H. (1976). The natural history of Meckel’s diverticulum and its relation to incidental removal. A study of 202 cases of diseased Meckel’s Diverticulum found in King County, Washington, over a fifteen year period. Am. J. Surg..

[4] Agha R.A., Borrelli M.R., Farwana R., Koshy K., Fowler A., Orgill D.P., For the SCARE Group (2018). The SCARE 2018 statement: updating consensus Surgical CAse REport (SCARE) guidelines. Int. J. Surg..

[5] Ueberrueck T., Meyer L., Koch A. (2005). The significance of Meckel’s diverticulum in appendicitis—a retrospective analysis of 233 cases. World J. Surg..

[6] Hansen C.C., Soreide K. (2018). Systematic review of epidemiology, presentation, and management of Meckel’s diverticulum in the 21st century. Medicine (Baltimore).

[7] Park J.J., Wolff B.G., Tollefson M.K. (2005). Meckel diverticulum: the Mayo Clinic experience with 1476 patients (1950–2002). Ann. Surg..

[8] Mendez-Garcia C., Suarez-Grau J.M., Rubio-Chaves C. (2011). Surgical pathology associated with Meckel s diverticulumin a tertiary hospital: 12 year review. Rev. Esp. Enferm. Dig..

[9] He Q., Zhang Y.L., Xiao B. (2013). Double-balloon enteroscopy for diagnosis of Meckel’s diverticulum: comparison with operative findings and capsule endoscopy. Surgery.

[10] Tseng Y.Y., Yang Y.J. (2009). Clinical and diagnostic relevance of Meckel’s diverticulum in children. Eur. J. Pediatr..

[11] Parvanescu A., Bruzzi M., Voron T., Tilly C., Zinzindohoue F., Chevallier J.M. (2018). Complicated Meckel’s diverticulum: presentation modes in adults. Medicine (Baltimore).

[12] Levy A.D., Hobbs C.M. (2004). From the archives of the AFIP. Meckel diverticulum: radiologic features with pathologic Correlation. Radiographics.

[13] Carmen Payá-Llorente, Gonzalo Garrigós-Ortega, Martínez-Pérez Aleix, Trullenque-Juan Ramón, Ernesto Amañanzas-Villena (2015). Giant Meckel’s diverticulum torsioned: an unnusual presentation. Rev. Esp. Enferm. Dig..

[14] Won Y., Lee H.W., Ku Y.M., Lee S.L., Seo K.J., Lee J.I. (2016). Multidetector-row computed tomography (MDCT) features of small bowel obstruction (SBO) caused by Meckel’s diverticulum. Diagn. Interv. Imaging.

[15] Varcoe R.L., Wong S.W., Taylor C.F., Newstead G.L. (2004). Diverticulectomy is inadequate treatment for short Meckel’s diverticulum with heterotopic mucosa. ANZ J. Surg..

